# Investigating the use of sensor-based IoET to facilitate learning for children in rural Thailand

**DOI:** 10.1371/journal.pone.0201875

**Published:** 2018-08-15

**Authors:** Pruet Putjorn, Panote Siriaraya, Farzin Deravi, Chee Siang Ang

**Affiliations:** 1 School of Engineering and Digital Arts, University of Kent, Canterury, Kent, United Kingdom; 2 Faculty of Industrial Design Engineering, Delft University of Technology, Delft, Netherlands; University of California, Berkeley, UNITED STATES

## Abstract

A novel sensor-based Internet of Educational Things (IoET) platform named OBSY was iteratively designed, developed and evaluated to support education in rural regions in Thailand. To assess the effectiveness of this platform, a study was carried out at four primary schools located near the Thai northern border with 244 students and 8 teachers. Participants were asked to carry out three science-based learning activities and were measured for improvements in learning outcome and learning engagement. Overall, the results showed that students in the IoET group who had used OBSY to learn showed significantly higher learning outcome and had better learning engagement than those in the control condition. In addition, for those in the IoET group, there was no significant effect regarding gender, home location (Urban or Rural), age, prior experience with technology and ethnicity on learning outcome. For learning engagement, only age was found to influence interest/enjoyment. The study demonstrated the potential of IoET technologies in underprivileged area, through a co-design approach with teachers and students, taking into account the local contexts.

## Introduction

The IoT (Internet of Things) is a new trend of information and communication technologies in which networked smart objects (termed “Things”) are able to exchange data over the internet [1]. Recently, this technology has been exploited in a variety of domains such as in healthcare, business, transportation and logistics [2]. In the field of education, it has been predicted that smart objects will become commonplace in the near future, providing students with access to rich and relevant learning content at a time of their convenience [3]. Through the use of networked smart tags, sensors and mobile computing devices, it is possible to transform everyday objects into interactive learning opportunities, allowing students to obtain authentic learning experiences from real world data [4]. The key advantages of IoT technology in such cases are to make the learning process more “real, local and fun”, allowing students to understand more complex concepts by making use of relevant information obtained from interaction with physical objects in the real world [3].

Although many studies investigating the use of IoET were carried out within the context of developed countries, such technology could also be beneficial in enhancing education in developing and underdeveloped regions as well. Prior studies have shown how similar technologies have been used successfully to help empower learning in developing nations [5–7]. The particular nature of an IoET-based learning environment could make it especially valuable in supporting education in rural and underdeveloped areas. Such technology has the potential to provide students with ubiquitous access to high quality learning content, hence helping reduce the inequality of learning performance [8]. However, implementing an IoET learning platform which relies too much on high-end electronic devices and commercial learning applications might be difficult due to the lack of financial resources and technological infrastructure. Furthermore, when implementing novel educational technologies in such an environment, a thorough understanding of the local context is needed [9]. Therefore, few studies to date have investigated how IoET technology could be effectively designed and used in such regions, particularly to support education at a primary school level.

The rural regions of Northern Thailand provide an interesting opportunity to investigate how IoET technology could be used to enhance the quality of education in underdeveloped areas. In such regions of Thailand, it has become a national priority to improve the skills and knowledge of students, especially in areas of mathematics and science [10] to help the country transition to a knowledge-based economy. Therefore, in an earlier government initiative, approximately 800,000 low-cost tablet computers (USD 30) were delivered to first-grade students across the kingdom as part of the One-Tablet-Per-Child Project (OTPC) to improve access to high-quality educational content [11]. However, this project was later discontinued due to unsatisfactory results. Numerous problems, such as the inability to integrate the educational content with the local curriculum and the lack of adequate technological infrastructure have been identified [12, 13].

**Fig 1:**
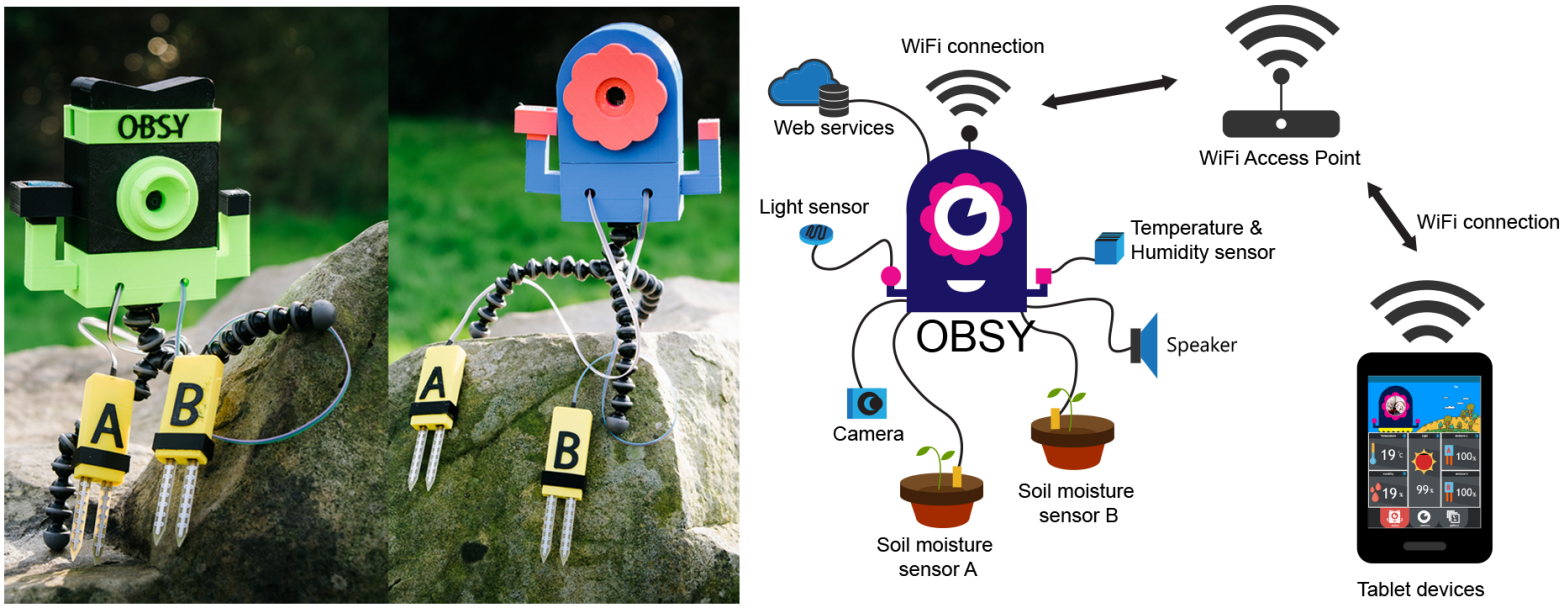
OBSY: Observation Learning System. OBSY device (left) and OBSY’s system diagram (right).

Against this backdrop, we aimed to design and develop a sensor-based IoET learning device to augment the existing OTPC mobile devices and support education in underdeveloped regions in Thailand. A 3-year study was carried out to investigate how such a device could be developed and used to effectively enhance learning outcome in science and maths for students in underprivileged areas in Northern Thailand. In the study, a sensor-based IoET prototype named OBSY (**Ob**servation Learning **Sy**stem) was developed which functions as a learning hub where students can access sensor data through wireless connection using their mobile tablet computers (Fig 1).

This paper focuses on the results from the evaluation study of OBSY which was carried out at four rural schools involving 244 students. A considerable number of students from these schools are of Akha ethnicity, a disadvantaged ethnic hill tribe who live in the highlands near the Thai border. Overall, the aims of this study are:

1. To investigate whether an IoET-based learning platform could help improve learning outcome in science and maths education for primary school students in rural areas.
2. To examine how factors related to the characteristic of the students (e.g. student demographics) could influence learning outcomes for students using an IoET-based learning platform.
3. To examine student preferences for future usage scenarios as well as desired functionalities of a sensor-based IoET learning platform.

## Materials and methods

As part of a 3-year project (Oct 2013- April 2016), an IoET prototype was iteratively designed and tested to support learning for primary school students in underdeveloped regions in Thailand. Throughout this period, various design workshops were carried out with both teachers and students to acquire feedback for the design and development of the prototype. In Oct 2013, a preliminary study was carried out to help us identify how an IoET learning system could be best designed to suit the local educational context. This was done by observing and interviewing students (*N* = 213) and teachers (*N* = 8) at both urban and rural schools about their use of mobile learning technology as part of the Thai government’s OTPC program.

The results from the preliminary study led to several design objectives which guided the initial development of our educational prototype [12, 13]. First, we discovered that there was a preference to provide students with a more active learning experience, one which enabled students to interact with information from the real world and obtain conceptual knowledge through a hands-on learning approach. The traditional teaching paradigms employed by Thai schools tend to focus on rote learning approaches (often due to the lack of teaching staff and scientific equipment needed to organize complex learning activities) and this was one aspect which the school staff would like to see changed. Secondly, due to the limited budget, a more affordable and flexible learning system was needed, one which could be more easily integrated into local lesson plans and the existing technological infrastructure. While tablet devices had already been distributed as part of the OTPC project, the school staff found it difficult to use them as part of their lesson plans, as the content offered on the tablets was too generalized and could not be easily customized (this resulted in them being used simply as e-book readers for existing text books). Finally, our initial studies showed that rural students in particular, tended to have higher anxiety when using technology in education and therefore, a more approachable and child friendly learning tool or device was needed.

Taking these findings into account, we designed and developed a sensor-based IoET prototype, named OBSY (Observation Learning System) (Fig 1 and Fig (a) in S1 Fig file). OBSY was designed to be used to support science-based learning activities at the primary school level. In order to appeal to young learners, OBSY was created to resemble a toy “octopus”, with the parts made using 3D printing technology. This anthropomorphic design was adopted to make the device more approachable for young children, hopefully helping reduce the level of anxiety during the learning activities. Various sensors were attached to OBSY and were designed to represent its “tentacles”. Through these sensors, users could use OBSY to collect information from their environment (such as the humidity, temperature and brightness). A mobile application was also created to allow students to access, store and interact with the information collected from the OBSY prototype and use that information as part of their science-based learning activities (See section “The final OBSY prototype” for more information).

**Fig 2:**
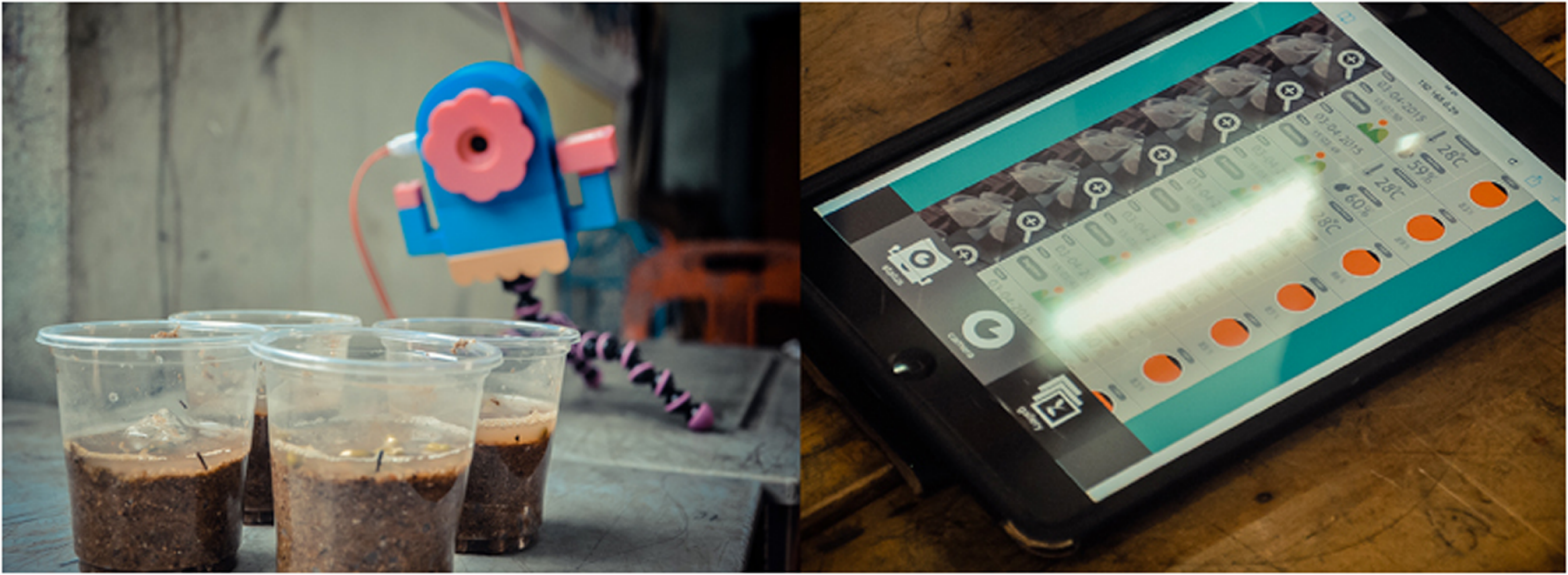
Students engaging with OBSY during the small-scale evaluation study.

Afterwards, a small-scale evaluation study (Fig 2) was carried out with the OBSY prototype in one urban and one rural school with 11 students and 2 teachers. This was done to evaluate the potential of OBSY in supporting learning and to identify any potential usability issues. Students and teachers were observed while using OBSY to carry out mock science experiments and later participants were interviewed to assess their learning engagement and overall experience in using OBSY. Based on their feedback, the OBSY prototype and lesson plans were further refined. In particular, the functionalities of OBSY were expanded. Two soil moisture sensors were added to allow students to simultaneously compare the differences between two observation subjects in various moisture conditions. In addition, the lesson plans were also revised to allow students to learn more advanced scientific concepts which better suit the schools’ educational curriculum (such as what factors are essential to plant growth etc.).

This refined prototype was then used to carry out a large scale evaluation study, the details of which are the main focus of this paper. The purpose of this evaluation study was to assess the effectiveness of the IoET based learning platform in schools located in underdeveloped areas. The study involved a total of 244 students and was carried out at four government supported schools in Chiang Rai. This paper focuses on the results from the quantitative evaluation. The results from the qualitative evaluation, based on the various interviews, observations and focus groups carried out during the study can be found at [14].

### The final OBSY prototype

The final version of OBSY was developed from a small single-board computer (The Raspberry Pi Model B+). (Fig 1). Various sensors (such as temperature, humidity, soil moisture and light intensity sensors) could be attached to OBSY, allowing it to gather information from the surrounding environment. A small video camera (designed to represent the “eye” of the octopus shaped OBSY) was also attached to the mainboard, allowing students to make visual observations from their environment. In addition, a web server was installed on OBSY, which hosted a web application that visually presented information from the sensors and camera. This enabled students to connect to OBSY wirelessly through their tablets without needing external internet connection. The web application was created using HTML 5 and JavaScript (Fig (a) in S2 Fig file) and contained three main screens. The status screen showed a live feed of the video from OBSY’s camera as well as data from the various sensors attached to OBSY. Using the camera screen, students were able to capture static images from the live video feed. The gallery screen showed a gallery of the previously captured images along with data about the environment when the image was captured (such as the date/time and temperature etc.) which is visualized through cartoon images.

### Evaluation study location

**Fig 3:**
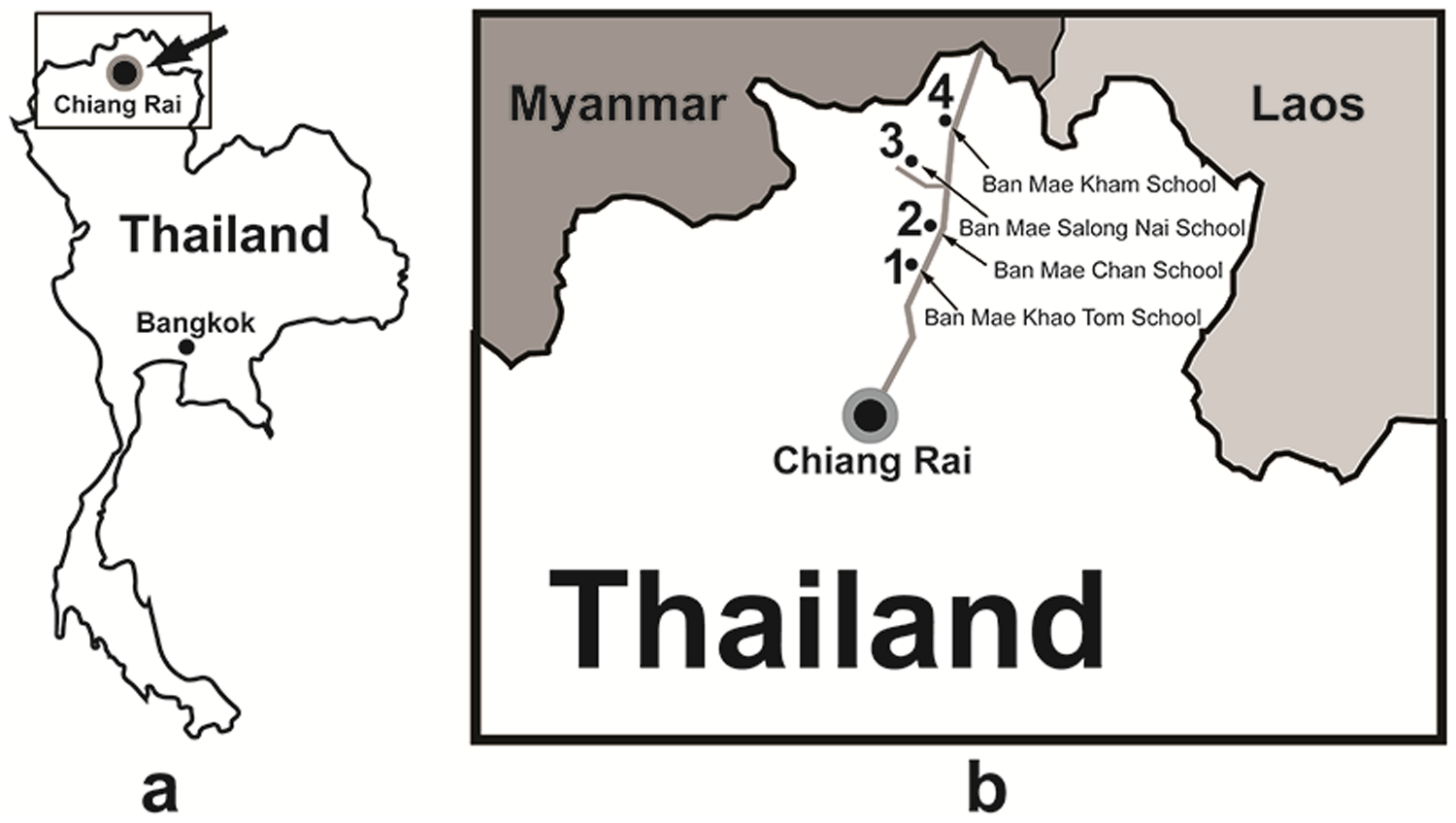
Study location map. Map of Chiang Rai, Thailand (a) and four participating rural schools (b).

The evaluation study was conducted at four rural schools located in Chiang Rai (Fig 3(a)) a city in the northernmost region of Thailand close to the border with Myanmar and Laos (Fig 3(b)). Chiang Rai has a population of 1.2 million and approximately 13% of the population are from hill tribes (eg. Karen, Hmong, Yao, Lahu, Akha and Lisu), a minority ethnic group living in the North of Thailand [15]. Fig 3(b) shows the location of the four underprivileged rural schools which took part in the final evaluation study. The first school was the Ban Mae Khao Tom School (Fig (a) in S3 Fig file), a government supported rural school approximately 45 km from the Thai and Myanmar border. Students from this school belong to hill tribes or are students of Thai ethnicity who come from lower income farming families. Such families live in remote areas where access to public services (infrastructure, education, health as well as administrative services) are often lacking [16]. The second school is the Ban Mae Chan School (Fig (b) in S3 Fig file), the largest school in the Mae Chan sub-district (about 30 km from the city of Chiang Rai). Most of the students are also tribal children. The third school is a small government supported school called Ban Mae Salong Nai (Fig (c) in S3 Fig file), located on the road to Doi Mae Salong, a mountain in Chiang Rai which was the centre of opium production in 1949. Students in this school are disadvantaged children who live with their families in the mountains. Their parents are mostly ethnic minority farm labourers who migrated from Myanmar. The school is reported to face a shortage of teaching staff, one teacher in each class had to teach all the subjects (maths, science, Thai, English, physical education etc.). The fourth school is a medium size school named Ban Mae Kham School (Fig (d) in S3 Fig file), located about 25 km from the Myanmar border. This school contains two classes at grade three, the majority of whom are also indigenous hill tribe children. Further information about the Thai education system can be found in supporting information (S1 Text).

### The evaluation study procedure

**Fig 4:**
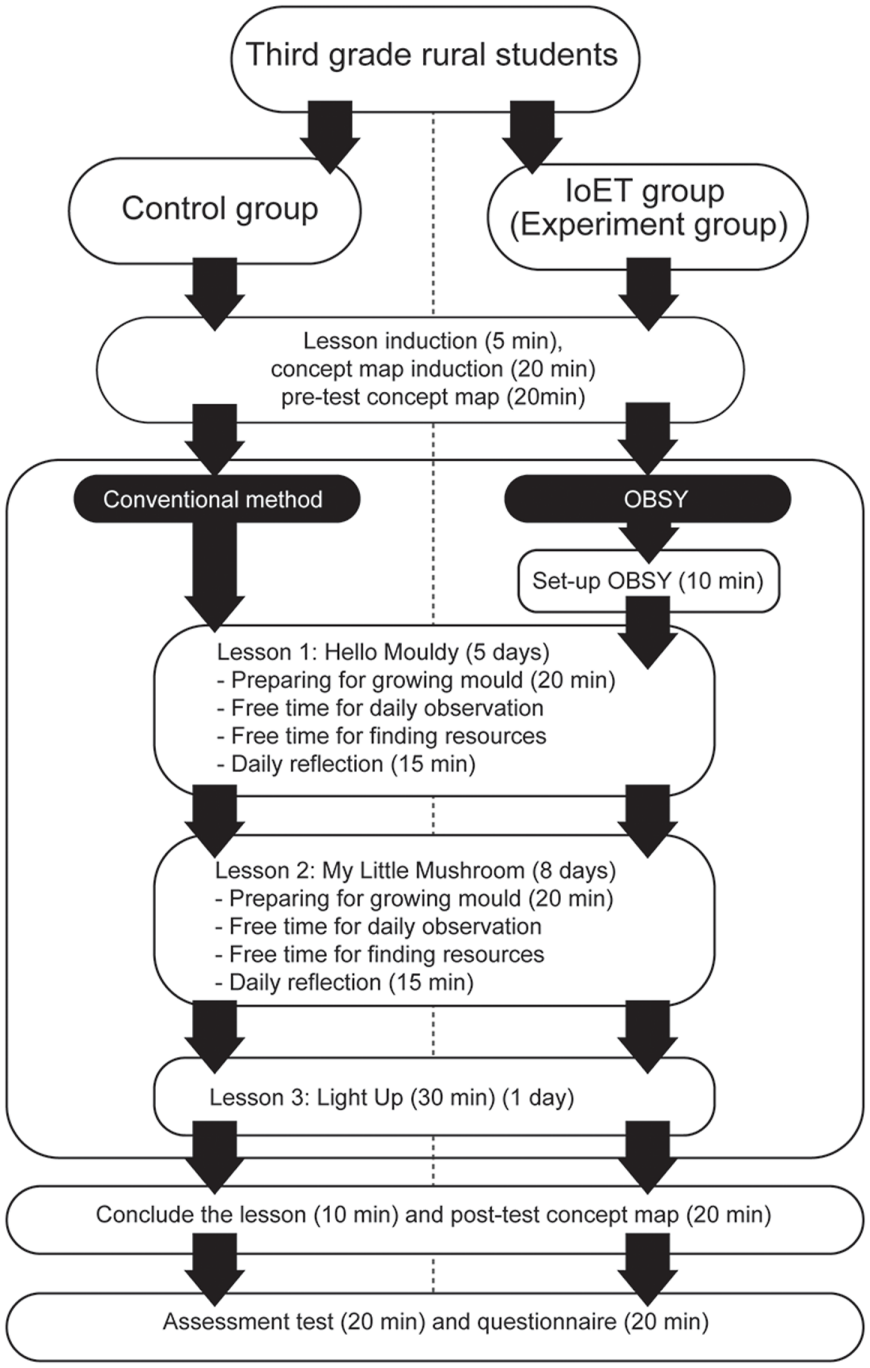
The experiment procedure.

The procedure used in the evaluation study is shown in Fig 4. Overall, this study lasted two weeks.

The students were first divided into a control group and an IoET group. Both groups followed the same procedure. Before the start of the science-based learning activities, a general introduction (e.g. about the research study and procedure) was given and the researcher introduced concept mapping to the students. To make sure students clearly understood how to create concept maps, they were first asked to practice by creating a concept map based on the ideas they were already familiar with (e.g. about plants). The students were then asked to create a concept map for the pre-test (Fig (a) in S4 Fig file). They were provided with blank paper and asked to draw a concept map demonstrating their prior knowledge about mushrooms.

The main experiment in the study consisted of three science-based learning activities which were related to the school curriculum:

1. “Hello Mouldy” (Fig (b) in S3 Fig file) introduced students to the study of mould and asked them to investigate how mould grows over a 5-day period in different conditions, by using either moist or dry breads and growing them in both bright and dark conditions. In this activity (Fig (a) in S7 Fig file), students in both groups typically started by preparing 6 pieces of bread and organised them into 3 sample groups (2 pieces per group). The first group was left in room conditions, while the second and third group were placed in darkroom conditions. Students took the third sample of the bread and sprinkled some water with a water sprayer to make it moist. They then took a piece of bread from each group, and sealed them in a clear re-sealable plastic bag. Students in IoET group took two pieces of the first sample bread, inserted the first OBSY moisture probe in the bread in the bag and sealed it properly while the second probe was inserted in the bread outside the bag. The OBSY hardware was placed carefully in front of the bread samples for observing the growing mould. Students could make observations at any free time during the day and used their tablets and OBSYs to observe real time video and capture images of growing mould while the control group recorded their observations without OBSY on the progress sheet.
2. In “My Little Mushrooms” (Fig (c) in S3 Fig file), students learnt about mushrooms by growing them in the same conditions as the first experiment to explore and identify what factors influenced their growth. The activity (Fig (b) in S7 Fig file) started with students organising 4 mushroom growing packs into 2 groups, with 2 packs in each group. One group was observed under bright room conditions and another group was monitored under darkroom conditions. Students then took 1 pack from each group and sprinkled some water with a water sprayer into the bottlenose of the growing pack to make it moist. Students in the IoET group took the mushroom growing packs, using the OBSY, inserted the first OBSY moisture probe in a dry plastic growing pack and another probe on a damp plastic growing pack. Two OBSYs were provided for each group, the first OBSY was used to observe growing mushrooms under bright room conditions, whereas another OBSY was used to observe inside the darkroom. Students could make observations at any free time during the day and used their tablets and OBSYs to observe real time video and captured images of growing mould while the Control group recorded their observation without OBSY on the progress sheet. During the learning activity of both groups (the control and IoET), the teachers encouraged them to learn through an inquiry-based learning approach, through the process of asking, investigating, creating, discussing and reporting. In addition, students in both groups were also allowed to make observations in their free time during the regular school day and were encouraged by teachers to learn more about the educational content (mushroom and related topics) by using the computer or tablet computer to access online learning contents (through YouTube etc.) or through the school reading materials.
3. In “Light Up” (Fig (d) in S3 Fig file), students explored how light travels and observed the amount of light which passes through various objects (transparent, translucent and opaque). A torch was used as a light source and was pointed towards various objects such as wax paper, plastic bag, fabric or wood (Fig (c) in S7 Fig file). Students rated and ordered the objects based on light transparency (how easily the object allows light to pass through). Students in both groups put the testing object in front of the light source. The OBSY group held the light source and pointed to the object and placed the OBSY’s light sensor as a screen to receive the amount of light which passed through the object. Students observed the amount of light on OBSY tablet application while the control group observed the amount of light without OBSY. Students in both groups then completed the objects observation sheet.

At the end of each day for both groups, the teacher carried out a short discussion and reflection session about the students’ findings. After the students had carried out all three learning activities, they were asked to draw the post-test concept map (Fig (e) in S3 Fig file). This was done to measure improvements in meaningful learning through comparison with the pre-test concept map. Then, the assessment tests were carried out to measure improvements in acquired content knowledge (Fig (f) in S3 Fig file). Finally, students in both groups were asked to complete the questionnaires for learning engagement and questionnaires to identify preferred usage scenarios and desired future functionalities for OBSY. The teacher read the questions out loud and the students filled in the questionnaires under the supervision of the teachers. This was done to make sure students understood the questions correctly as some students had low skills in reading and writing.

### Participants

A total of 244 children and 8 teachers took part in the evaluation study. Grade three students (Prathom 3) were recruited from the 4 rural schools. In each school, 4 groups of 8 students (4 boys and 4 girls) were selected. Students in the control condition carried out science-based learning activities by recording their findings using pen and paper, whilst students in the IoET condition were asked to use OBSY as an observational tool to carry out the same science-based learning activity. Overall a total of 116 students participated in the OBSY condition and 128 students participated in the control condition. In addition, two teachers in each school with experience in using the tablet computers were recruited to help facilitate the science-based learning activities. Ethical approval was granted by the University’s Research Ethics Advisory Group to carry out research with young students. Informed consent was granted by each school’s director. Written consent was also provided on behalf of the parents including permission for audio and video recording.

### Measurements

**Fig 5:**
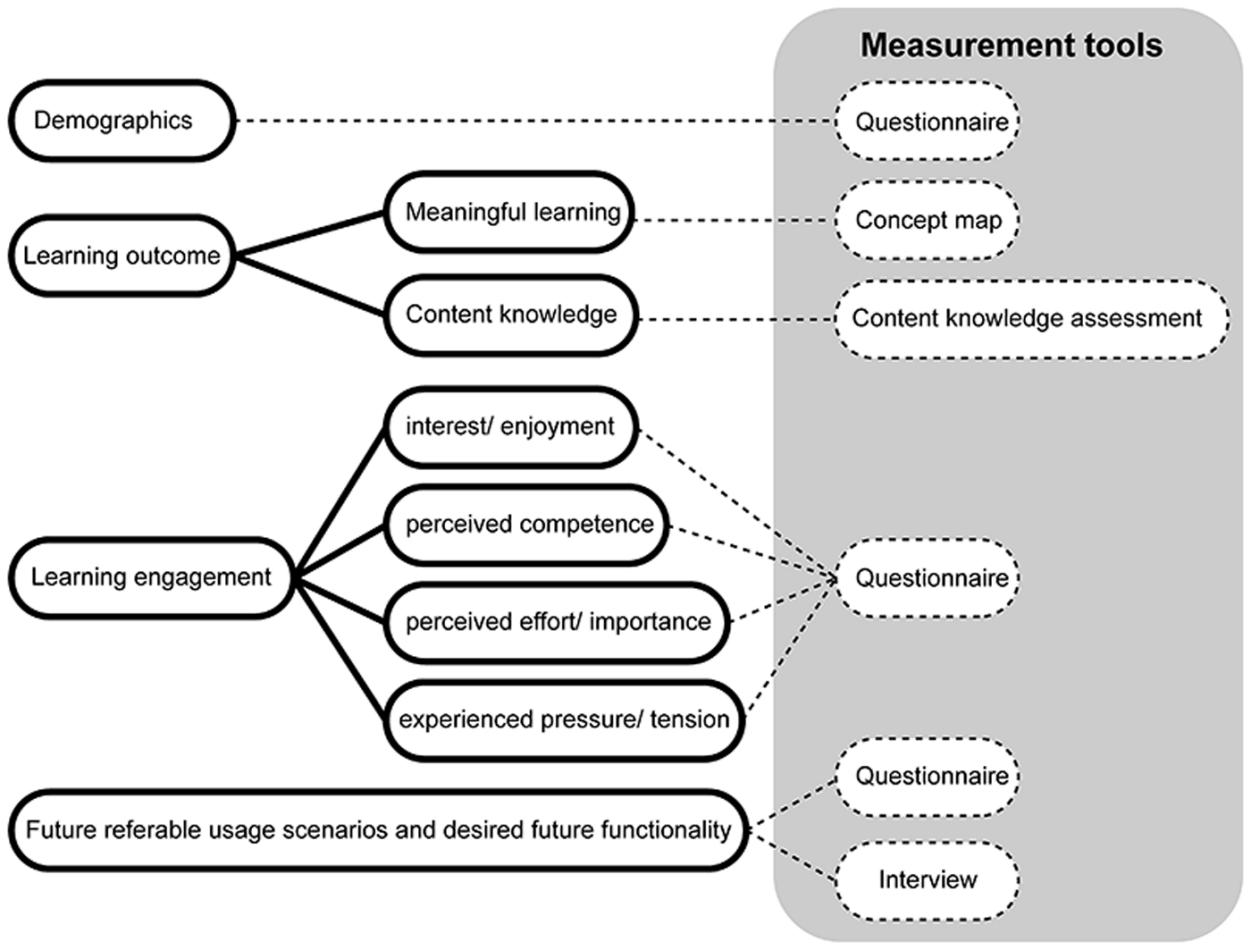
A summary of the measurement tools.

Fig 5 provides a summary of the measurements used in this study. Overall, educational outcome was measured mainly through two categories of variables, Learning outcome and Learning engagement. Participants were also asked to provide basic demographical information (such as age, gender, ethnicity and house location) and information about their prior technology experience (the usage of internet, computer, tablet computer and mobile devices) [17]. In addition, participants were asked to rate different future scenarios in which they felt OBSY could be used to enhance learning as well as different functionalities and features which participants wanted to see in the future.

#### Learning outcome measurements

Learning outcome was measured mainly through the concept of meaningful learning and from the acquired *content knowledge*. Meaningful learning refers to how learners are able to learn and construct new knowledge and concepts to solve specific problems by linking together their previous relevant knowledge. Such a concept has *“an emphasis on constructivism and hands-on inquiry-oriented instruction to promote children’s conceptual knowledge by building on prior understanding, active engagement with the subject content, and applications to real world situations”* [18].

To assess meaningful learning, pre-tests and post-tests were carried out with concept maps (Fig (a) in S5 Fig file) which is widely acknowledged as an effective tool to assess the students’ critical thinking skills, cognitive process and performance [19]. Concept maps have been used in prior research studies involving science and mobile learning to measure scientific literacy [20] and assess the students’ learning and performance [21]. The scoring criteria used in this study was based on Novak [22], in which the concept map were scored based on the component and structure, one point was assigned for each valid proposition, five points for each level of hierarchy, one point for each branch, ten points for each valid cross-link and one point for any specific examples.

An assessment test was carried out to measure the acquired content knowledge. Content knowledge refers to knowledge and information about a specific topic that is learnt [23]. It should be noted that only a post-test of content knowledge was performed as we needed to reduce excessive pressure on students and avoid creating a barrier to the learning activities due to performance anxiety [24]. This assessment (Fig (a) in S6 Fig file) was aimed at measuring content knowledge through 2 topics of science (temperature and plant growth conditions) and 3 topics of mathematics which students needed to understand to carry out scientific experiments (percentage, fraction and ordering/sequencing numbers). The questions used in the assessment were reviewed by the teachers and tested in a pilot with representative students.

#### Learning engagement measurements

To measure learning engagement, questionnaires were administered to students (see S1 Appendix for the full questionnaires). The questionnaires were modified from the Intrinsic Motivation Inventory (IMI) [25–27] and the level of tension represented how anxious students felt when asked to carry out the learning activity [28–30]. Four variables were measured: i) perceived interest and enjoyment, ii) perceived competence, iii) perceived effort/importance and iv) the level of pressure/tension.

#### Preferable usage scenarios and desired future functionality of OBSY

Students in the IoET group were also asked to rate different scenarios in which they wanted to see OBSY being used in the future to enhance learning as well the desired future functionality of OBSY. This would allow us to understand more from the design and development perspective, about the broader context in which OBSY-like IoET technology could be used to support learning in the future as well as provide a guideline for designing an effective learning tool to improve educational outcome for students in underdeveloped regions. The learning scenarios and design features in this questionnaire were formulated based on feedback from interviews carried out during the small-scale testing session as part of the iterative design process and was based on the concept of Mobile Seamless Learning Environments [31]. The different future scenarios include i) using IoET as a learning companion, ii) using IoET in a broader array of subjects (apart from science and mathematics learning) iii) using IoET to carry out experiments outside the classroom context (i.e. for pervasive learning), iv) adding a real-world social element, allowing OBSY to be used as a communication device to talk to friends and family while learning. In addition, participants were asked to rate the different functionalities and design features which they desired to see in OBSY in the future. These included: i) having audio and voice functionality, ii) having the ability for ubiquitous connectivity (observations could be carried out anytime, anyplace, far away from the device), iii) mobility, being able to navigate around the environment by itself, iv) DIY customizability (students are able to optimise and customise the OBSY device by themselves These aspects were rated on a 5-point Likert scale.

## Results

### Participant demographics

In total, 244 third grade students and 8 teachers from 4 schools participated in the study. The demographic profile of the students is shown in Table 1.

**Table 1:** Demographic profiles of students from the control and IoET group.

Items	Control group	IoET group	Total
	N (%)	N (%)	N (%)
N	116	128	244
Age (Min 7, Max 17)	*M* = 9.08(*SD* = 1.522)	*M* = 10.16(*SD* = 2.075)	*M* = 9.64(*SD* = 1.907)
**Gender**			
Girl	54 (43.9%)	69 (56.1%)	123 (50.4%)
Boy	62 (51.2%)	59 (48.8%)	121 (49.6%)
**Ethnic**			
Thai	40 (56.3%)	31 (43.7%)	71 (29.1%)
Other	76 (43.9%)	97 (56.1%)	173 (70.9%)
**Home location**			
Urban	27 (44.3%)	34 (55.7%)	61 (25%)
Rural	89 (48.6%)	94 (51.4%)	183 (75%)
**School**			
Ban Mae Khao Tom	32 (50%)	32 (50%)	64 (26.2%)
Ban Mae Chan	32 (49.2%)	33 (50.8%)	65 (26.6%)
Ban Mae Salong Nai	31 (45.6%)	37 (54.4%)	68 (27.9%)
Ban Mae Kham	21 (44.7%)	26 (55.3%)	47 (19.3%)

Overall, there were an almost equal numbers of boys (49.6%) and girls (50.4%) of whom 29.1% were Thai and another 70.9% were of ethnic minority (tribal students). The majority of them lived in rural areas (75%). It should be noted that the students’ age range of 7 to 17 years (*M* = 9.64) is higher than the national standard (8-9 years old). This is because for some hill tribe students who came from border countries such as Myanmar or Laos, schooling has been delayed. When such students join the Thai school system, they have to start a new education process at the primary level (year one) as a result of their lack of Thai language proficiency in writing, speaking and communication. The smaller participant numbers from the fourth school (Ban Mae Kham School) was due to the fact that it had only one grade three class. Overall, statistical tests showed that there were no significant differences between the control and the experiment group in gender (*X*^2^ = 1.317, *p* < .251), ethnicity (*X*^2^ = 3.107, *p* < .078) and home location (*X*^2^ = .351, *p* < .554). However, there was a significant difference (*t*(242) = 8.960, *p* < .001) between both groups in age where the IoET group (*M* = 9.08, *SD* = 1.522) was approximately one year older than the control group (*M* = 10.16, *SD* = 2.075). As such, this aspect was controlled for when carrying out statistical testing to examine differences in learning outcome between both groups.

When examining the prior level of technology experience (which was calculated from the frequency of technology ownership and usage [17]), the results showed that approximately 85% of students (in both the control and the IoET condition) have used a computer or a tablet computer before. More students have tablet computers at home (48.4%) compared to desktop computers (37.3%). The majority have access to mobile phones (90.6%) whereas only 32% have Internet access at home. The results of an independent-samples t-test showed that Thai students had significantly higher technology experience compared with ethnic minority students (*t*(242) = 8.96, *p* < .001) and urban students had significantly higher technology experience compared to rural students (*t*(242) = 4.37, *p* < .001). However, there was no significant differences in technology experience between genders (*t*(242) = 0.71, *p* < .476) (See Table 2). The tests also showed that there was a significant difference between the control and IoET group, where students in the IoET group had higher technology experience than the control group (*t*(242) = 2.51, *p* < .013). As such, this aspect was also controlled for examining differences in learning outcome between both groups.

**Table 2:** Demographic profiles of students from the control and IoET group.

Items	Group		Gender		Ethnicity		Home location	
	control	IoET	Boy	Girl	Thai	Other	Urban	Rural
	*M*(*SD*)	*M*(*SD*)	*M*(*SD*)	*M*(*SD*)	*M*(*SD*)	*M*(*SD*)	*M*(*SD*)	*M*(*SD*)
Technology	3.54	4.05	3.87	3.74	4.99	3.32	4.49	3.58
Experience	(1.781)	(1.285)*	(1.512)	(1.606)	(1.281)	(1.397)**	(1.362)	(1.556)**

*Significant at *p* < .05.,

** Significant at *p* < .01.

### The differences in learning outcome between the control and IoET group

Independent-samples t-tests were conducted to compare the differences in learning outcome between students in both groups. The results showed that there was a significant difference between the pre-test concept map scores (*M* = 7.60, *SD* = 4.80) and post-test concept map scores (*M* = 17.10, *SD* = 7.41) for the control condition (*t*(115) = 15.83, *p* < 0.001). Similarly there was also a significant difference between the pre-test concept map scores (*M* = 13.77, *SD* = 7.84) and post-test concept map scores (*M* = 32.73, *SD* = 11.86) for the IoET condition (*t*(127) = 26.44, *p* < 0.001). As such, the results suggested that there was meaningful improvement in learning after students participated in the learning activities in both conditions.

When examining the pre-test concept map scores between the control and IoET group however, t-tests also showed that there was a significant difference in the pre-test concept map scores between students in the IoET condition (*M* = 7.60, *SD* = 4.80) and the control condition (*M* = 13.77, *SD* = 7.84), (*t*(242) = 7.32, *p* < .001). This is likely due to the nature of the student recruitment process in this study. To avoid disrupting the structure of the lessons and putting too much strain on the limited number of teaching staff available, the school directors decided to assign the students to the IoET and control condition as a group based on their class (instead of separating the students from their class and dividing them into smaller groups to carry out the learning activities, which would then need to be supervised and taught independently). As such, the directors decided to randomly select which class would participate in the IoET and control condition learning activities. As there tended to be inequality in student performance between the classes in the rural schools, students who participated in the control condition turned out to have lower pre-test concept map scores than those in the IoET condition.

Therefore, to control for the confounding effects of prior knowledge, as well as prior technology experience and age (aspects found to be significantly different between the IoET and control group), a two stage hierarchical multiple regression analysis was carried out to test the differences in learning outcome between the two groups, with improvement in meaningful learning (calculated from the difference between the post-test and pre-test concept map scores) as the dependent variable [32]. The collinearity statistics (i.e. Tolerance and VIF) were all within accepted limits and the assumption of multicollinearity was met. Pre-test concept map scores, age and prior technology experience were included in the first stage of the regression to control for their effects. The condition in which students carried out the learning activity was entered in the second stage of the regression. The results are shown in Table 3.

**Table 3:** Regression analysis to measure improvements in meaningful learning.

Variables	Δ*R*^2^	*B*	*SEB*	*β*
Model 1	.067			
(Constant)		11.568	3.739	
Pre-test concept map		.293	.081	.243**
Age		.109	.325	.024
Technology experience		-.350	.378	-.062
Model 2	.260			
(Constant)		18.396	3.260	
Pre-test concept map		.055	.073	.046
Age		-.586	.286	-.128
Technology experience		-1.129	.332	-.201**
Learning condition (control (0) vs IoET (1))		10.321	1.074	.590**

*Significant at *p* < .05.,

** Significant at *p* < .01.

The hierarchical multiple regression analysis showed that at stage one, a significant regression equation was found (*F*3, 240 = 5.728, *p* < .001) and accounted for 6.7% of the variation. Only pre-test concept map scores contributed significantly to the prediction (*β* = .243, *p* < .001). The addition of the learning condition in the second stage explained an additional 26% of the variation (*F*4, 239 = 29.04, *p* < .001). Both technology experience (*β* = −.201, *p* < .001) and the learning condition (*β* = .590, *p* < .001) were significant predictors of scores in meaningful learning. This suggested that the learning condition had significant influence on scores in meaningful learning even when controlling for pre-test scores, technology experience and age. Overall, students in the IoET group had higher improvement in meaningful learning (*M* = 18.96, *SD* = 8.11) in comparison to the control group (*M* = 9.5, *SD* = 6.46).

To assess improvements in educational outcome as measured from the overall content knowledge which students acquired after the learning activities, another hierarchical multiple linear regression analysis was carried out. The overall content knowledge assessment scores were a summation of the scores in the different knowledge categories (temperature, plant growing condition, percentage, fraction and ordering/sequencing numbers). Age, pre-test concept map scores and prior technology experience were used as control variables. The results of the regression analysis are shown in Table 4.

**Table 4:** Regression analysis to measure improvements in acquired content knowledge.

Variables	Δ*R*^2^	*B*	*SEB*	*β*
Model 1	.128**			
(Constant)		9.613	2.074	
Pre-test concept map		.131	.045	.190**
Age		.553	.181	.210**
Technology experience		.725	.210	.225**
Model 2	.096**			
(Constant)		11.996	2.009	
Pre-test concept map		.048	.045	.070
Age		.310	.176	.118
Technology experience		.454	.204	.141*
Learning condition (control (0) vs IoET (1))		3.602	.661	.359**

*Significant at *p* < .05.,

** Significant at *p* < .01.

Pre-test concept map scores, age and technology experience contributed significantly to the regression model (*F*3, 240 = 11.70, *p* < 001) and accounted for 12.8% of the variation. The addition of the learning condition in the second stage explained an additional 9.6% of the variation (*F*4, 239 = 17.24, *p* < .001). The results showed that both prior technology experience (*β* = 0.141, *p* < .05) and the learning condition which the students participated in (*β* = .359, *p* < .001) were significant predictors towards the content knowledge which students acquired after the learning activities. Overall, students in the IoET group (*M* = 21.25, *SD* = 3.28) showed higher learning outcome as measured from acquired content knowledge when compared to the control groups (*M* = 16.79, *SD* = 5.56).

In addition, hierarchical multiple regression analysis was also conducted to observe the students’ achievement of content knowledge in each category. Once again, age, technology experience and pre-test concept map score were entered as control variables and each category of content knowledge (knowledge about temperature, plant/mushroom growing, fraction, percentage and ordering/sequencing) was used as the predictor variable. Tables of these results can be found in supporting information (S1 Table).

In stage one, a significant regression equation was found for the dependent variables temperature (*F*3, 240 = 14.421, *p* < .001, *R*^2^ = 15.3%), plant growth knowledge (*F*3, 240 = 5.695, *p* < .001, *R*2 = 6.6%), fraction (*F*3, 240 = 2.641, *p* < .050, *R*^2^ = 3.2%) and ordering & sequencing numbers (*F*3, 240 = 8.082, *p* < .001, *R*^2^ = 9.2%). However, a significant regression equation was not found for percentage (*F*3, 240 = 0.112, *p* < .953, *R*2 = 0.1%). The addition of the learning condition (control vs IoET group) in the second stage explained an additional 22.8% of the variation in temperature (*F*4, 239 = 17.596, *p* < .001), 20.7% of the variation in plant growing condition (*F*4, 239 = 15.599, *p* < .001), 7.9% of the variation in Fraction (*F*4, 239 = 5.125, *p* < .001) and 10.1% of the variation in ordering & sequencing numbers (*F*4, 239 = 6.682, *p* < .001).

For content knowledge about the temperature, the results showed age (*β* = .227, *p* < .001) and the learning condition (*β* = .316, *p* < .001) to be significant predictors. Technology experience (*β* = .128, *p* < .046) and learning condition (*β* = .434, *p* < .001) were found to be significant predictors for plant growth knowledge. For fraction, the only learning condition (*β* = .251, *p* < .001) was found to be a significant predictor. For ordering & sequencing numbers, pre-test scores (*β* = .142, *p* < .043) and age of students (*β* = .162, *p* < .026) were significant predictors, but not the learning condition.

Overall, the regression analysis indicated that students in the IoET group showed higher learning outcome in content knowledge about the temperature (OBSY (*M* = 4.39, *SD* = 0.896), control (*M* = 2.82, *SD* = 2.257)), plant growing condition (OBSY (*M* = 4.045, *SD* = 0.827), control (*M* = 2.47, *SD* = 2.199)), and fraction (OBSY (*M* = 3.91, *SD* = 1.165), control (*M* = 3.28, *SD* = 1.456)).

### The difference in learning engagement between the control and IoET condition

Prior studies in education [25, 33] found academic success to be significantly related to the learning engagement. Therefore, in this study, the differences in learning engagement between the control and IoET condition were also measured. Hierarchical multiple regression analyses were conducted to predict the effect of the learning condition on learning engagement based on four different variables (interest and enjoyment, perceived competence, effort/importance and pressure/tension). Age, technology experience and the pre-test concept map score were entered as control variables and the learning condition as the predictor variable (S2 Table). In stage one, a significant regression equation was found for the dependent variables interest/ enjoyment (*F*3, 240 = 2.80, *p* < .041, *R*^2^ = 3.4%), effort/importance (*F*3, 240 = 2.954, *p* < .033, *R*^2^ = 3.6%) and pressure/ tension (*F*3, 240 = 4.497, *p* < .004, *R*^2^ = 5.3%). The addition of the learning condition in the second stage explained an additional 3.1% of the variation in interest/enjoyment (*F*4, 239 = 4.157, *p* < .003), 9.2% of the variation in competence (*F*4, 239 = 6.362, *p* < .001), 6.9% of the variation in effort/ importance (*F*4, 239 = 6.996, *p* < .001) and 14.4% of the variation in pressure/ tension (*F*4, 239 = 14.649, *p* < .001). For interest/enjoyment, the results showed age (*β* = −.224, *p* < .001) and the learning condition (*β* = .204, *p* < .001) to be significant predictors. Similarly, for effort/ importance, both age (*β* = −.189, *p* < .01) and the learning condition (*β* = .304, *p* < .001) were also found to be significant predictors. However, for competence, only the learning condition (*β* = .351, *p* < .001) was found to be a significant predictor. The learning condition (*β* = −.432, *p* < .001) was also the only significant predictor for pressure/tension. Overall, the regression results indicated that students in the IoET group showed higher interest/enjoyment (OBSY (*M* = 4.717, *SD* = 0.401), control (*M* = 4.65, *SD* = 0.511)), competence (OBSY (*M* = 4.395, *SD* = 0.545), control (*M* = 4.05, *SD* = 0.613), and effort/ importance (OBSY (*M* = 4.084, *SD* = 0.682), control (*M* = 3.677, *SD* = 0.773)). Students also showed lower pressure/tension in the IoET group (*M* = 1.78, *SD* = 0.814) compared with the control group (*M* = 2.56, *SD* = 0.915).

### The effect of student demographics on learning engagement and learning outcome for the IoET condition

The previous section showed that using OBSY improved students’ learning performance and engagement. In this section, we wanted to know if demographic factors are related to the learning outcome and engagement measures for the group using the sensor-based IoET device. Therefore, multiple regression analysis was carried out only for students in the OBSY group, with each of the learning outcome measures (meaningful learning (calculated from the difference between the post-test and pre-test concept map scores) and content knowledge) and each of the learning engagement measures (interest/enjoyment, perceived competence, effort/importance and pressure/tension) as dependant variables, while age, gender, ethnicity, home location and technology experience were entered as independent variables for each of the regression analysis. The collinearity statistics (i.e. Tolerance and VIF) were all within accepted limits and the assumption of multicollinearity was met. Overall, a significant regression model was not found for content knowledge and meaningful learning. For the learning engagement measures, a significant regression model was found for interest/enjoyment ($F\left(5,122\right)=3.40,p<.007,R^{2}=.122,R_{Adjusted}^{2}=.086$) where interest/ enjoyment could be predicted from the age of students (*β* = −.402, *p* < .001) (S2 Table). For Perceived competence, effort/importance and pressure/tension a significant regression model was not found.

### Preferable future usage scenarios and desired features and functionalities of OBSY

Students were asked to rate what design factors they found desirable for a sensor-based learning platform and how they wished to use such a technology in future learning. Table 5 shows the overall mean scores. Most of the preferred usage scenarios and desired features and functionalities were highly rated.

**Table 5:** Regression analysis to measure improvements in acquired content knowledge.

Category	Items	Mean	SD
Prefer future usage scenario	Learning companion (I want OBSY to be a friend)	4.58	1.009
	Additional learning subjects (I want to use OBSY in a broader array of subjects)	4.10	1.309
	Pervasive learning (I want to use OBSY for outdoor experiments)	4.56	1.018
	Additional social element (I want OBSY to be used as a communication device)	4.26	1.275
Desired features and functionalities	Ubiquitous connectivity feature (I want to use OBSY anywhere)	4.23	1.233
	Mobility functionality (I want OBSY to move/walk)	4.40	1.125
	Audio functionality (I want OBSY to have sound/voice)	4.61	.618
	DIY Customizability feature (I want to build my own OBSY)	4.41	1.147

Repeated measured ANOVA tests were also conducted to determine whether there were any differences in the scores between the “preferable future usage scenarios” and “desired features and functionalities”. The results showed that there was not a significant difference in the scores for the “desired features and functionalities”. For the scores in the “preferable future usage scenarios”, the repeated measures ANOVA with a Greenhouse-Geisser correction showed that there was a significant difference (*F*(2.815, 354.496) = 8.976, *p* < 0.01). Post hoc tests using the Bonferroni correction revealed that the mean score of “additional learning subjects” (*M* = 4.10, *SD* = 1.31) was significantly different to the other preferable usage scenarios: learning companion (*M* = 4.58, *SD* = 1.00, *p* < 0.01), pervasive learning (*M* = 4.56, *SD* = 1.01, *p* < 0.001) and additional social element (*M* = 4.61, *SD* = .618, *p* < 0.01).

## Discussion

### The effect of IoET technology on learning outcome and engagement

A number of studies have shown that mobile and IoET-based technology could be used to help improve learning for students in classroom settings [5–7]. The results from this study suggest that such technology could be useful in improving educational outcome in underdeveloped areas as well. Compared to prior studies, the current study was based on rural schools in Thailand, where the technology infrastructure was often obsolete and students generally had less experience and more anxiety towards computer technology than their urban counterparts [17]. The results from this study show that students from such schools who had used OBSY to carry out the scientific learning activities had higher improvements in meaningful learning (as measured by changes in concept map score) and acquired content knowledge when compared to a control condition.

We believe that higher outcomes in both meaningful learning and content knowledge acquisition in the OBSY group could be explained by a number of factors. The rich information feedback features available from the OBSY learning platform could have contributed to the improved learning outcome. For instance, the observational and recording ability of OBSY’s “eye camera” which allows students to observe physical objects in various stages and record that information could have contributed to a better assimilation (learners reflect on knowledge after observation) and accommodation (learners modify their existing knowledge based on the observations) of new knowledge during the learning activities. More specifically, the ease with which OBSY could be used to make observations of the plants and mushrooms at various stages of growth, augmented with real-time sensory data could have enabled students to better conceptualize their scientific knowledge about plant growth. This is similar to using videos and images to augment the learning content and help improve learning outcome [34].

Another aspect which could have contributed to the higher learning outcome was the rich hands-on learning experience from the students’ interactions as they used OBSY to observe various objects during the learning activities and manipulated the variables of the environment to understand their effects. In the “Light Up” activity, for example, we observed two students learn about light transmission, in which one student explained the concept to the other student in their local language and blocked the light sensor attached to OBSY to show the other student how light transmission worked in practice. In addition, another key advantage of this platform which was mentioned during the interviews was that OBSY allowed students to learn from examples local to their environment. In the “Hello Mouldy” learning activity, students were able to learn about fungus life cycles not through generic examples of fungi from textbooks or online videos, but by experimenting with fungi frequently found in their environment. At the same time, students were able to physically experience the effect of the scientific concepts (such as time, temperature etc.) on the objects, experiment and manipulate them, observe, label and compare those phenomena through the virtual graphical representations of those concepts on the tablet application. For example, students were taught about the concept of time by first seeing a visualization of it on the OBSY program (time was visualized through an analog clock and the concept of day and night was represented with a background image of a mountain which was bright during the day and dark during the night). Then, students learned more about time by manipulating the timer to tell OBSY when photos of the plants should be taken. Finally, they were able to associate the concept of time passing with plant growth by comparing various photographs of the plants taken at different intervals as time passed.

Overall, this ability of OBSY to provide students with the opportunity to obtain conceptual knowledge from authentic hands-on learning tasks was one of the main advantages of this system, as described by the teachers during the interview:

“It [OBSY] helps them to see the real world rather than watching video clips from YouTube. Video clips from YouTube have been used in the past to show plants growing and animal life cycles but they lack a connection to the students because the situations and environment are different.”

(44 year old, female, teacher).

“I think that students are able to learn about concepts such as percentages and concepts such as temperature better in a natural setting, by actually experiencing those concepts. Such experimental style education is not commonly used [in Thai Schools]”

(55 years old female teacher).

Prior studies have argued that such a hands-on learning approach could help students make sense of the scientific concepts more easily, contributing to their scientific learning [35]. Overall, this could explain why the results showed that students using OBSY demonstrated higher acquisition of content knowledge of the two science-related topics measured (temperature, plant/mushroom growth).

For mathematics related topics, students using OBSY showed higher learning outcome on the topic “Fractions”. This may be due to how real world data is visualized on the OBSY mobile app, through the presentation of dynamic visual objects. For instance, a picture of the sun was used to visualize the concept of brightness and was “filled up” based on the current brightness of the environment (for example, a fully filled sun was presented to represent bright environments, while a half-filled sun was used to show darker environments (Fig (a) in S2 Fig file). Such visual representations combined with immediate and real-time feedback have been found to help students to understand concepts [36]. Regular checking for an update of the sensor information based on such graphical visualisations might have therefore contributed to their better understanding of fractions.

Previous studies have shown that using technology such as mobile or tablet computers in education could result in an increase in students’ learning engagement, which can influence their learning outcome [37–40]. However, other studies have also found that the misuse of such technology in the classroom could disengage students and hinder learning (cited in Rossing [41]). Our results indicated that students using IoET technology demonstrated higher learning engagement in all four categories compared to the control group. Specifically, students who used OBSY had higher interest/enjoyment and less pressure/tension. We suspect this could be due to the anthropomorphic design of OBSY. The playful nature of this design, similar to what is found in smart toys [42, 43] helped to provoke curiosity and approachability for children and thus could have an effect in reducing anxiety and tension during learning [44, 45]. Interviews with teachers and students about OBSY carried out after the learning activities supported this notion, as they reported that the form factor of OBSY, shaped as a cartoon character, combined with the doll-like appearance helped draw in students which in turn motivated them to engage with OBSY (see [14] for more information). This they believed, was one factor which helped encourage the students to put more effort into the learning activities. Students in the interviews also often mentioned that they would like the learning interactions to be presented through such human-like attributes (i.e. if OBSY were able to “tell” them the temperature in a conversation-like manner)(see [14]).

“*The children liked OBSY and the equipment was attractive to the students. They kept asking what this equipment does… The curiosity towards OBSY draws in crowds of students*.”

(24 year old, male, teacher).

“*Children were always interested in the form/shape of OBSY… they were excited and wanted to take part due to their curiosity*.”

(25 year old, female, teacher)

However, further experimental studies would need to be carried out to provide a more definite conclusion as to the effect of these attributes on learning engagement.

### The influence of student demographics

Previous studies have shown that factors such as gender, age and technology experience could influence the technology mediated learning outcome [46–48]. As such, these factors were examined in this study. Furthermore, as this study focused on underdeveloped areas, the effect of home location (whether students lived in rural areas or in the urban) and ethnicity was examined. Prior studies found that disparities between rural and urban education and ethnicity in Thailand have resulted in imbalanced economic and social development [49] and thus educational inequalities [50]. The results showed that Thai students and those living in urban areas had significantly higher computer experience than their counterparts. This was not surprising, as a number of studies investigating the acceptance of ICT technology in Thailand show that there still remains a digital divide in Thailand, due to inadequate government support in ICT infrastructure and services in rural areas [51, 52]. Interestingly, gender, ethnicity, prior experience with technology and home location did not influence the learning outcome or the learning engagement for students in the IoET group. In other words, regardless of these factors, students in the IoET group benefitted equally well from using OBSY. The results from this study also showed that older students reported less interest/engagement. This could be explained by the anthropomorphic design of OBSY which resembled a toy octopus. While this design could draw in interest and appeal from younger students and thus help improve their engagement with the learning activities, older students in the OBSY group might have “out-grown” such features and therefore reported less interest/enjoyment [53]. Overall, these results are encouraging, as it suggests that in the OBSY group, students could equally benefit from using this technology to enhance learning, regardless of their background.

### Preferable usage scenarios and desired features and functionalities of OBSY

As there has been limited research exploring how IoET technology could be developed to improve educational outcome for students in underdeveloped regions, it is challenging for designers to envisage different scenarios in which such technology could be used effectively. During the iterative design phase of OBSY, various interviews and user testing sessions were carried out which identified a number of future scenarios in which OBSY could be used. For example, one frequent suggestion was to use OBSY to extend the traditional classroom context, allowing students to create their own learning environment at their free time, in a way relevant to their daily environment (such as collaboratively through their local communities).

“I think that OBSY could be useful for outdoor learning context in encouraging students’ curiosity to use it to gain new knowledge. Students passionately use OBSY to see the mushrooms after their lunch time.”

(25 years old, female, teacher).

Overall, most of the future usage scenarios and desired functionalities were rated highly. Such scenarios and desired functionalities were often mentioned during the interviews conducted after the main study where for example students reported wanting to take OBSY home to observe local farm animals and pets or wanting to use OBSY to learn English (in addition to math and science) by having OBSY speak with them in English.

Student interests in similar usage scenarios and desired features have also been reported in previous studies. For instance, one study showed that outdoor learning experiences could improve learning engagement [54] and another study proposed the value of hybrid learning scenarios, where playful activity is combined with outside classroom learning [55]. Finally, the interest in audio functionality may be due to the interest of this group in engaging with learning content in multimedia formats [56].

## Conclusion

We believe that several contributions have been made in the study. First, we have contributed to the design and development of a sensor based IoET system and have provided an example of how such a system could be embedded as part of a blended learning activity for primary school children. The design of this system, which involved an anthropomorphic aspect and was designed to provide a hands-on experience in relevant science-based learning activities, evolved through an iterative design process. This design process took into account the feedback of teachers and students, both in the design of the IoET device and in the selection of the learning content and design of the corresponding blended learning activity. The results and knowledge from this design process might be valuable to those looking to deploy similar technological systems to enhance learning for young children. In addition, as a contribution to the field of Human Computer Interaction For Development (HCI4D)(which seeks to understand more about how people and computers interact in developing regions and how such systems can be designed specifically for these contexts), we have shown how IoET technology could be used to support education in underprivileged areas. Prior studies have highlighted the problems in educational outcome for students living in underprivileged areas and those from ethnic minority groups [45, 51]. As such, there is often a national interest to improve the learning performance of students in such areas to help reduce education inequality [57]. Although, technology such as e-learning or mobile based learning could be introduced to improve educational outcome in this context [58–60], various factors could hinder such efforts. Our results showed that an anthropomorphized IoET device, when used as part of blended learning activities to provide authentic learning experiences, was able to improve learning outcome, both in terms of meaningful learning, acquired content knowledge and learning engagement. Furthermore, the IoET device was built based on an existing mobile learning platform (The OTPC tablet computers) that had been widely distributed and used in Thailand. The technological aspects were specifically designed to suit the local context, such as allowing the device to function in areas where internet is unavailable or otherwise unstable. Finally, the results from the evaluation in this study adds to our existing repertoire of knowledge, showing how mobile and IoET technology could be used to enhance learning, as well as examining the implications of student demographics for those using IoET technology.

There are a number of limitations regarding the generalizability of this study. First, the study focused on science-related learning activities that were carried out within the classroom context. In addition, the study also focused on primary school children in Northern Thailand. Whether the results would still be applicable for students in a different age group, geographical context or for different learning subjects would still need to be investigated. In the future, we are particularly interested in examining how this technology could be used to provide authentic and hands-on learning experiences to help teach more advance concepts in other subjects (such as in social science or history). In addition, it would be interesting to examine whether such technology could be valuable when used outside a structured classroom learning context, particularly, in local communities to allow students to acquire relevant learning experiences in daily life.

## Supporting information

S1 Text The education system in Thailand. (DOCX)

S1 Fig OBSY concept sketch. (TIF)

S2 Fig Screenshots of the web application. (TIF)

S3 Fig Pictures of the four rural schools. Ban Mae Khao Tom School (a), Ban Mae Chan School (b), Ban Mae Salong Nai School (c) and Ban Mae Kham (d).
(TIF)

S4 Fig Pictures of the experiment procedure. (a) A pre-test with the concept map, (b) Hello Mouldy experiment, (c) My Little Mushrooms experiment, (d) Light Up experiment, (e) A post-test concept map, (f) Content knowledge assessment and questionnaires.
(TIF)

S5 Fig Pictures of students drawing a concept map. (TIF)

S6 Fig Assessments used to measure acquired content knowledge. (TIF)

S7 Fig The experiment activities. (TIF)

S1 Table Regression analysis of the achievement of students’ learning performance about temperature, plant growing conditions, percentage, fraction and ordering & sequencing numbers. (DOCX)

S2 Table Regression analysis of students’ interest/ enjoyment for IoET condition. (DOCX)

S1 Appendix Questionnaires. (DOCX)
