# Supplementary material for: Investigating the use of sensor-based IoET to facilitate learning for children in rural Thailand
Source: PLoS One. 2018 Aug 15;13(8):e0201875. doi: 10.1371/journal.pone.0201875 (PMC6093682; doi:10.1371/journal.pone.0201875)
Supplement: S1 Text — (DOCX) [file pone.0201875.s001.docx]

**The Education system in Thailand.**

The basic Thai education system is divided into six years of primary education and six years of secondary education. The government has an obligation to provide free education during these twelve years of learning. There are four key stages including Prathom 1-3 (the first stage of primary school for 7-9 year-old children), Prathom 4-6 (the second stage of primary school for 10-12 year-old children), Matthayom 1-3 (low secondary education for children aged 13-15) and Matthayom 4-6 (upper secondary education for children aged 15-17). Participants in this study consisted of primary school children. At the primary school level, eight core subjects are compulsorily taught in school, including the Thai language, mathematics, science, social science, health and physical education, arts and music, technology and foreign languages (primarily English).
