# Supplementary figures and images for: Investigating the use of sensor-based IoET to facilitate learning for children in rural Thailand
Source: PLoS One. 2018 Aug 15;13(8):e0201875. doi: 10.1371/journal.pone.0201875 (PMC6093682; doi:10.1371/journal.pone.0201875)

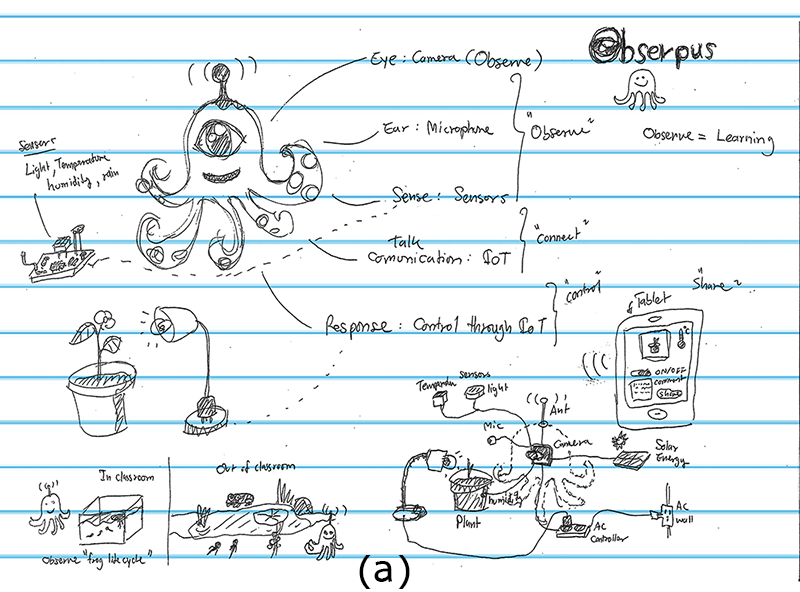

Supplement: S1 Fig — (TIF) [file pone.0201875.s002.tif]

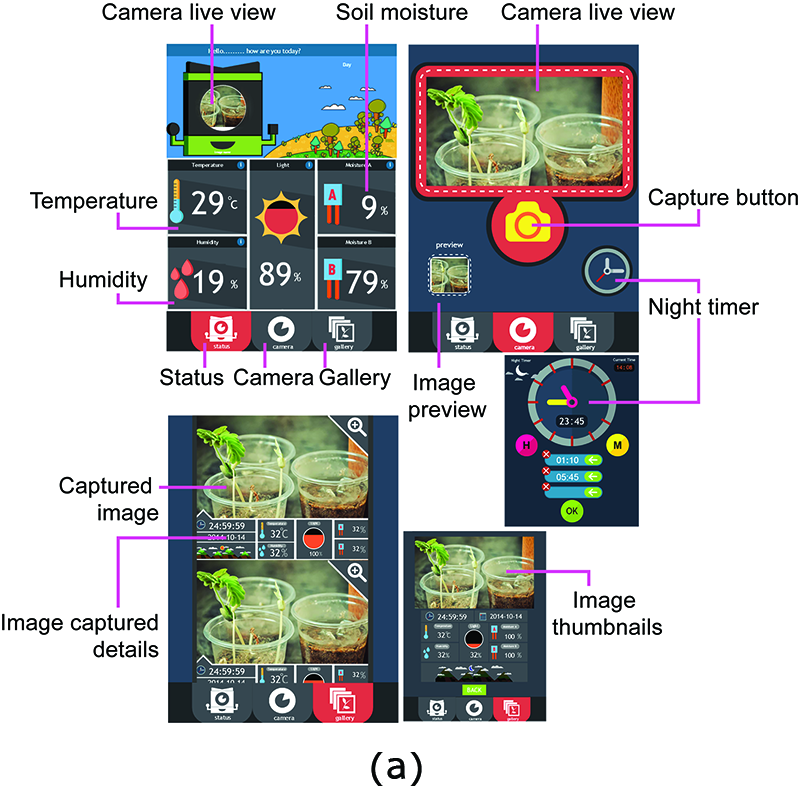

Supplement: S2 Fig — (TIF) [file pone.0201875.s003.tif]

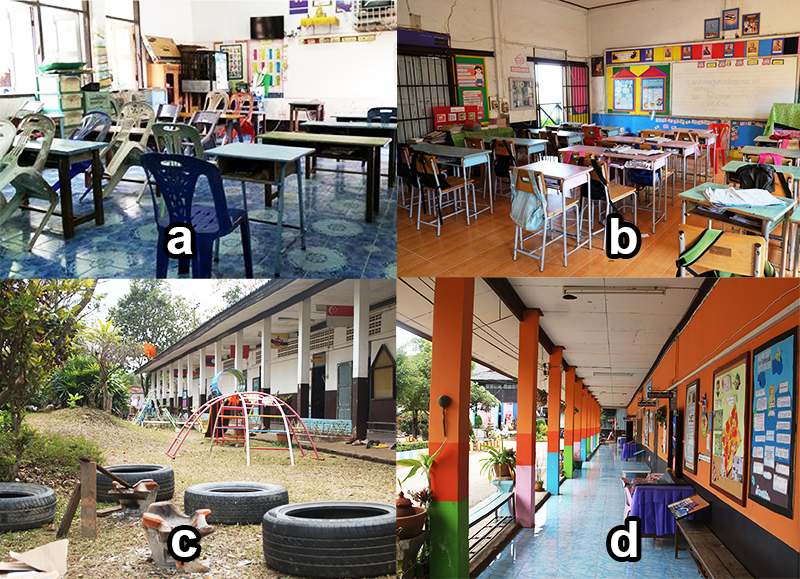

Supplement: S3 Fig — Ban Mae Khao Tom School (a), Ban Mae Chan School (b), Ban Mae Salong Nai School (c) and Ban Mae Kham (d). (TIF) [file pone.0201875.s004.tif]

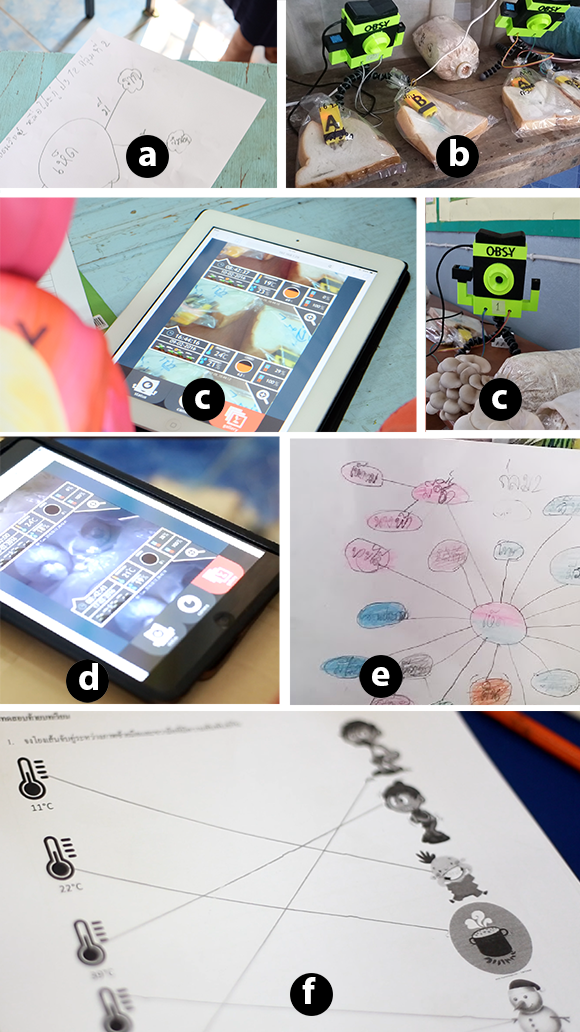

Supplement: S4 Fig — (a) A pre-test with the concept map, (b) Hello Mouldy experiment, (c) My Little Mushrooms experiment, (d) Light Up experiment, (e) A post-test concept map, (f) Content knowledge assessment and questionnaires. (TIF) [file pone.0201875.s005.tif]

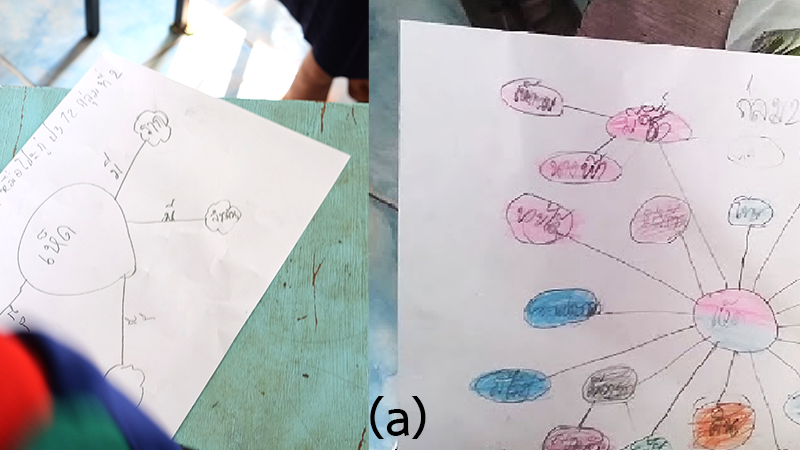

Supplement: S5 Fig — (TIF) [file pone.0201875.s006.tif]

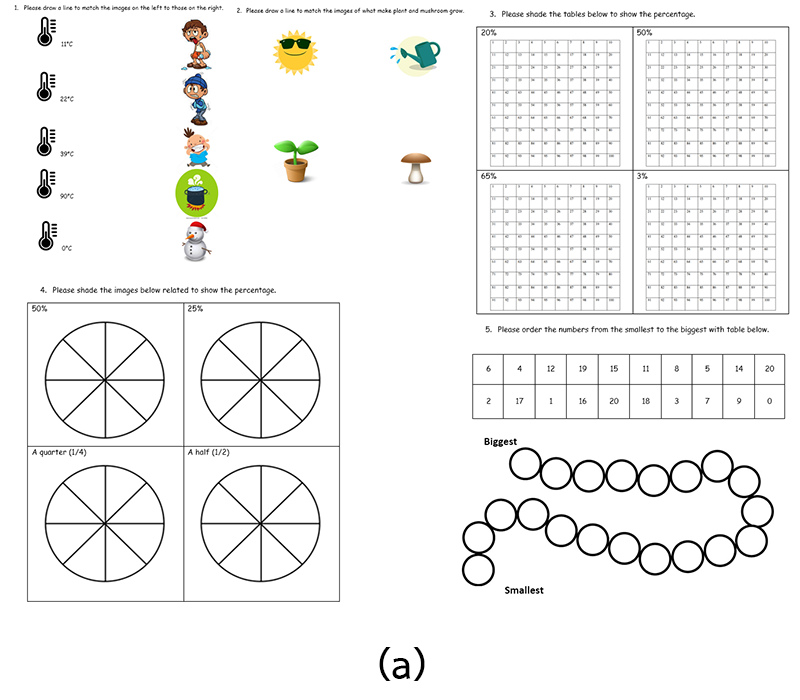

Supplement: S6 Fig — (TIF) [file pone.0201875.s007.tif]

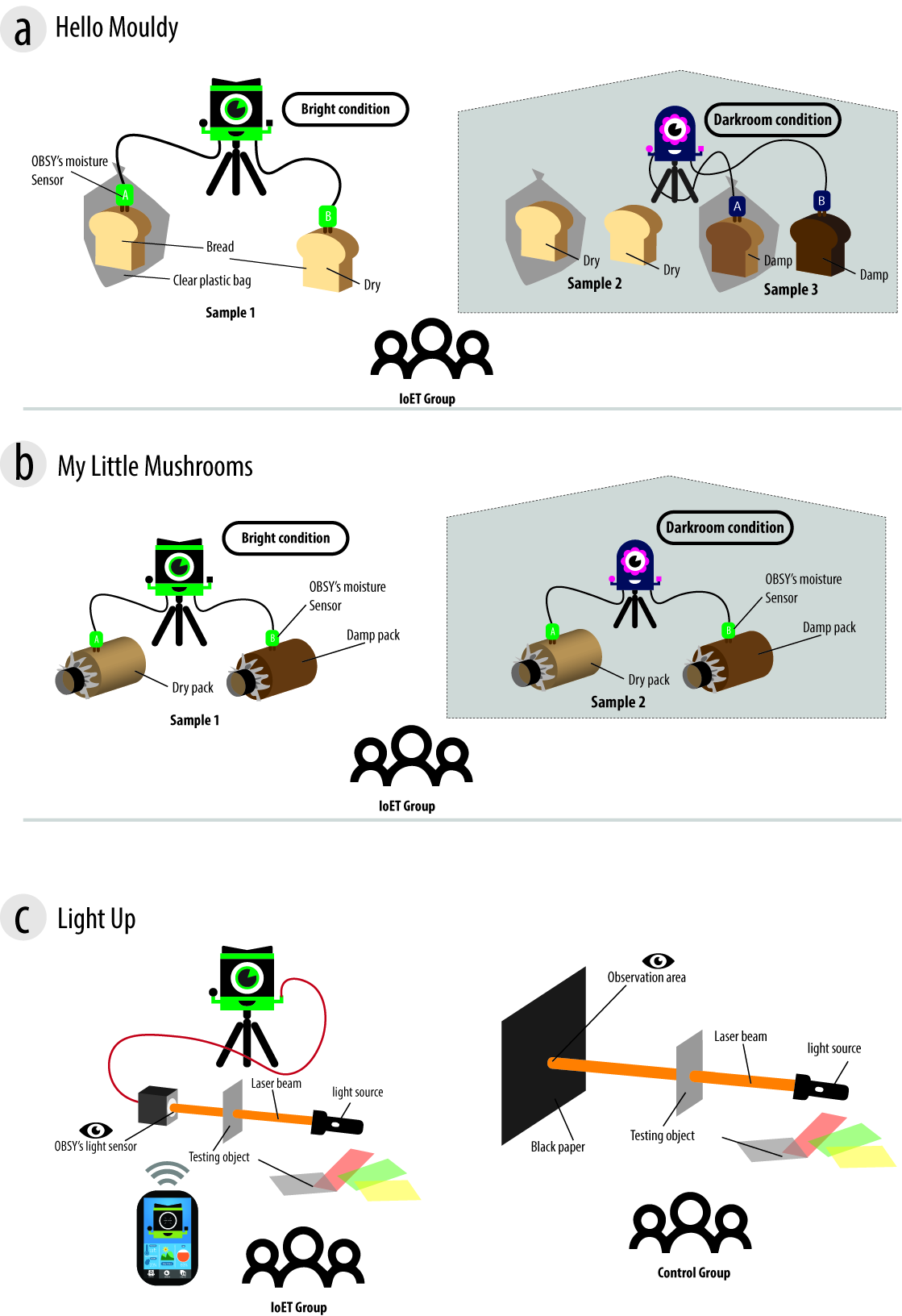

Supplement: S7 Fig — (TIF) [file pone.0201875.s008.tif]
