# Supplementary material for: Investigating the use of sensor-based IoET to facilitate learning for children in rural Thailand
Source: PLoS One. 2018 Aug 15;13(8):e0201875. doi: 10.1371/journal.pone.0201875 (PMC6093682; doi:10.1371/journal.pone.0201875)
Supplement: S1 Table — (DOCX) [file pone.0201875.s009.docx]

**Regression analysis of the achievement of students’ learning performance about temperature, plant growing conditions, percentage, fraction and ordering**

Regression analysis of the achievement of students’ learning performance about temperature and plant growing conditions.

| Variables | Temperature | | | |  | Plant growing condition | | | |
| --- | --- | --- | --- | --- | --- | --- | --- | --- | --- |
|  | Δ*R^2^* | *B* | *SE B* | *β* |  | Δ*R^2^* | *B* | *SE B* | *β* |
| Model 1 | .153** |  |  |  |  | .066** |  |  |  |
| (Constant) |  | -.524 | .756 |  |  |  | .743 | .772 |  |
| Pre-test concept map |  | .043 | .016 | .167** |  |  | .020 | .017 | .079 |
| Age |  | .300 | .066 | .309** |  |  | .137 | .067 | .145* |
| Technology experience |  | .212 | .076 | .178** |  |  | .267 | .078 | .230** |
| Model 2 | .075** |  |  |  |  | .141** |  |  |  |
| (Constant) |  | .252 | .741 |  |  |  | 1.779 | .731 |  |
| Pre-test concept map |  | .016 | .017 | .061 |  |  | -.016 | .016 | -.065 |
| Age |  | .221 | .065 | .227** |  |  | .032 | .064 | .033 |
| Technology experience |  | .123 | .075 | .104 |  |  | .149 | .074 | .128* |
| control (0) vs IoET (1) |  | 1.174 | .244 | .316** |  |  | 1.567 | .241 | .434** |
| *Significant at *p*<.05. ** Significant at *p*<.01. | | |  |  |  |  |  |  |  |

Regression analysis of the achievement of students’ learning performance about percentage and fraction.

| Variables | Percentage | | |  | | | Fraction | | | |
| --- | --- | --- | --- | --- | --- | --- | --- | --- | --- | --- |
|  | Δ*R^2^* | *B* | *SE B* | | *β* |  | Δ*R^2^* | *B* | *SE B* | *β* |
| Model 1 | .001 |  |  | |  |  | .016* |  |  |  |
| (Constant) |  | 4.799 | .456 | |  |  |  | 3.449 | .586 |  |
| Pre-test concept map |  | .003 | .010 | | .023 |  |  | .020 | .013 | .108 |
| Age |  | -.022 | .040 | | -.041 |  |  | -.046 | .051 | -.066 |
| Technology experience |  | -.014 | .046 | | -.021 |  |  | .103 | .059 | .119 |
| Model 2 | .008 |  |  | |  |  | .008** |  |  |  |
| (Constant) |  | 4.657 | .466 | |  |  |  | 3.896 | .587 |  |
| Pre-test concept map |  | .008 | .010 | | .057 |  |  | .005 | .013 | .024 |
| Age |  | -.008 | .041 | | -.014 |  |  | -.092 | .052 | -.130 |
| Technology experience |  | .003 | .047 | | .004 |  |  | .052 | .060 | .060 |
| control (0) vs IoET (1) |  | -.215 | .153 | | -.104 |  |  | .675 | .193 | .251** |
| *Significant at *p*<.05. ** Significant at *p*<.01. | | |  | |  |  |  |  |  |  |

Regression analysis the achievement of students’ learning performance about ordering and sequencing numbers.

| Variables | Ordering & sequencing numbers | | | | |
| --- | --- | --- | --- | --- | --- |
|  | Δ*R^2^* | *B* | *SE B* | *β* |  |
| Model 1 | .092** |  |  |  |  |
| (Constant) |  | 1.142 | .779 |  |  |
| Pre-test concept map |  | .045 | .017 | .177** |  |
| Age |  | .185 | .068 | .190** |  |
| Technology experience |  | .158 | .079 | .133* |  |
| Model 2 | .009** |  |  |  |  |
| (Constant) |  | 1.415 | .796 |  |  |
| Pre-test concept map |  | .036 | .018 | .140 |  |
| Age |  | .157 | .070 | .162* |  |
| Technology experience |  | .127 | .081 | .107 |  |
| control (0) vs IoET (1) |  | .412 | .262 | .112 |  |
| *Significant at *p*<.05. ** Significant at *p*<.01. | |  |  |  |  |
