# Supplementary material for: Investigating the use of sensor-based IoET to facilitate learning for children in rural Thailand
Source: PLoS One. 2018 Aug 15;13(8):e0201875. doi: 10.1371/journal.pone.0201875 (PMC6093682; doi:10.1371/journal.pone.0201875)
Supplement: S2 Table — (DOCX) [file pone.0201875.s010.docx]

Regression analysis of students’ interest & enjoyment

| Variables | Interest/enjoyment | | | |  |
| --- | --- | --- | --- | --- | --- |
|  | Δ*R^2^* | *B* | *SE B* | *β* |  |
| Model 1 | .034* |  |  |  |  |
| (Constant) |  | 5.184 | 0.199 |  |  |
| Pre-test concept map |  | -0.003 | 0.004 | -0.048 |  |
| Age |  | -0.041 | 0.017 | -0.171** p<.018 |  |
| Technology experience |  | -0.019 | 0.020 | -0.063 |  |
| Model 2 | .031** |  |  |  |  |
| (Constant) |  | 5.308 | 0.201 |  |  |
| Pre-test concept map |  | -0.007 | 0.004 | -0.117 |  |
| Age |  | -0.054 | 0.018 | -0.224** p<.003 |  |
| Technology experience |  | -0.033 | 0.020 | -0.111 |  |
| Learning condition (control (0) vs IoET (1)) |  | 0.187 | 0.066 | 0.204** p<.005 |  |
