# Supplementary material for: Investigating the use of sensor-based IoET to facilitate learning for children in rural Thailand
Source: PLoS One. 2018 Aug 15;13(8):e0201875. doi: 10.1371/journal.pone.0201875 (PMC6093682; doi:10.1371/journal.pone.0201875)
Supplement: S1 Appendix — (DOCX) [file pone.0201875.s011.docx]

**Questionnaire for students**

**Please answer the following questions.**

**1. Section A: Personal Information**

1.1 Your age……………………… 1.2 You are a: □ Boy □ Girl

1.3 What is your nationality? □ Thai □ Other

1.4 Where do you live? □ Urban areas □ Rural areas

**2.** **Section B; My technology experience**

| 2.1 Do you have the internet at home? | □ Yes | □ No |
| --- | --- | --- |
| 2.2 Do you have a tablet at home? | □ Yes | □ No |
| 2.3 Do you have a computer at home? | □ Yes | □ No |
| 2.4 Do you have a smart phone at home? | □ Yes | □ No |
| 2.5 Have you used a tablet computer before? | □ Yes | □ No |
| 2.6 Have you used a computer before? | □ Yes | □ No |

2.7 How long do you spend on the internet a week? ……………………………hour/s

2.8 How long do you spend on a computer a week? ……………………………hour/s

2.9 How long do you spend on a tablet computer a week? ……………………………hour/s

**3. Section C; How I use the OBSY system (usability test)**

| **Questions** | **Strongly disagree** |  |  |  | **Strongly agree** |
| --- | --- | --- | --- | --- | --- |
|  |  | **** | **** | **** |  |
|  | **1** | **2** | **3** | **4** | **5** |
| 3.1 I would like to use OBSY every day. |  |  |  |  |  |
| 3.2 I found OBSY make me confuse. (R) |  |  |  |  |  |
| 3.3 I thought OBSY was easy to use. |  |  |  |  |  |
| 3.4 I would need my teacher to help me to use OBSY. (R) |  |  |  |  |  |
| 3.5 I found the instructions easy to follow |  |  |  |  |  |
| 3.6 I thought there was too much error about OBSY. (R) |  |  |  |  |  |
| 3.7 I think most of my friends would learn to use OBSY very quickly. |  |  |  |  |  |
| 3.8 I found OBSY difficult to use. (R) |  |  |  |  |  |
| 3.9 I felt very confident using OBSY. |  |  |  |  |  |
| 3.10 I needed to learn a lot of things before I could get going with OBSY. (R) |  |  |  |  |  |

**4. Section D; What I think about this learning activity (learning engagement)**

| **Questions** | **Strongly disagree** |  |  |  | **Strongly agree** |
| --- | --- | --- | --- | --- | --- |
|  |  | **** | **** | **** |  |
|  | **1** | **2** | **3** | **4** | **5** |
| **4.1 Interest/Enjoyment** |  |  |  |  |  |
| 4.1.1 I enjoyed doing this activity very much. |  |  |  |  |  |
| 4.1.2 This activity was fun to do. |  |  |  |  |  |
| 4.1.3 I thought this was an exciting activity |  |  |  |  |  |
| 4.1.4 This activity held my attention very well |  |  |  |  |  |
| 4.1.5 I would describe this activity as very interesting. |  |  |  |  |  |
| **4.2 Perceived Competence** |  |  |  |  |  |
| 4.2.1 I think I am pretty good at this activity. |  |  |  |  |  |
| 4.2.2 After working at this activity for a while‚ I felt pretty competent. |  |  |  |  |  |
| 4.2.3 I am satisfied with my performance at this activity. |  |  |  |  |  |
| 4.2.4 I was pretty skilled at this activity. |  |  |  |  |  |
| 4.2.5 This was an activity that I couldn’t do very well. (R) |  |  |  |  |  |
| **4.3 Effort/Importance** |  |  |  |  |  |
| 4.3.1 I put a lot of effort into this. |  |  |  |  |  |
| 4.3.2 I didn’t try very hard to do well at this activity. (R) |  |  |  |  |  |
| 4.3.3 I tried very hard on this activity. |  |  |  |  |  |
| 4.3.4 It was important to me to do well at this activity. |  |  |  |  |  |
| 4.3.5 I didn’t put much energy into this. (R) |  |  |  |  |  |
| **4.4 Pressure/Tension** |  |  |  |  |  |
| 4.4.1 I did not feel nervous at all while doing this. (R) |  |  |  |  |  |
| 4.4.2 I felt very tense while doing this activity. |  |  |  |  |  |
| 4.4.3 I was very relaxed doing this activity. (R) |  |  |  |  |  |
| 4.4.4 I was anxious while working on this activity. |  |  |  |  |  |
| 4.4.5 I felt pressured while doing this activity. |  |  |  |  |  |

**Questionnaire for teacher**

**Please answer the following questions.**

**1. Section A: Personal Information**

Your age……………………… You are a: □ male □ female

**2.** **Section B; My technology experience**

| 2.1 Do you have the internet at home? | □ Yes | □ No |
| --- | --- | --- |
| 2.2 Do you have a tablet at home? | □ Yes | □ No |
| 2.3 Do you have a computer at home? | □ Yes | □ No |
| 2.4 Do you have a smart phone at home? | □ Yes | □ No |
| 2.5 Have you used tablet computer before? | □ Yes | □ No |
| 2.6 Have you used a computer before? | □ Yes | □ No |

2.7 How long do you spend on the internet a week? ……………………………hour/s

2.8 How long do you spend on a computer a week? ……………………………hour/s

2.9 How long do you spend on a tablet computer a week? ……………………………hour/s

**3. Section C; How I use the OBSY system for my teaching (usability test)**

| **Questions** | **Strongly disagree** |  |  |  | **Strongly agree** |
| --- | --- | --- | --- | --- | --- |
|  |  | **** | **** | **** |  |
|  | **1** | **2** | **3** | **4** | **5** |
| 3.1 I think that I would like to use OBSY frequently. |  |  |  |  |  |
| 3.2 I found OBSY complex. |  |  |  |  |  |
| 3.3 I thought OBSY was easy to use. |  |  |  |  |  |
| 3.4 I think that I would need the support of a technician to be able to use OBSY. |  |  |  |  |  |
| 3.5 I found the various functions were well integrated. |  |  |  |  |  |
| 3.6 I thought there was too much inconsistency in the OBSY system. |  |  |  |  |  |
| 3.7 I would imagine that most my colleagues would learn to use OBSY very quickly. |  |  |  |  |  |
| 3.8 I found OBSY very cumbersome to use. |  |  |  |  |  |
| 3.9 I felt very confident using OBSY. |  |  |  |  |  |
| 3.10 I needed to learn a lot of things before I could get going with OBSY. |  |  |  |  |  |
